# Use and benefit of information, communication, and assistive technology among community-dwelling older adults – a cross-sectional study

**DOI:** 10.1186/s12889-023-16926-8

**Published:** 2023-10-13

**Authors:** Marina L. Fotteler, Thomas D. Kocar, Dhayana Dallmeier, Brigitte Kohn, Sarah Mayer, Ann-Kathrin Waibel, Walter Swoboda, Michael Denkinger

**Affiliations:** 1 DigiHealth Institute, Neu-Ulm University of Applied Sciences, Wileystrasse 1, 89231 Neu-Ulm, Germany; 2 Institute for Geriatric Research, Ulm University Medical Center, Ulm, Germany; 3 Agaplesion Bethesda Clinic Ulm, Ulm, Germany; 4 Department of Epidemiology, Boston University School of Public Health, Boston, USA; 5 Geriatric Center Ulm, Ulm, Germany

**Keywords:** Information and communication technology, Assistive technology, Benefit, Sociodemographic, Older adults, Cross-sectional

## Abstract

**Background:**

Technology can support healthy aging and empower older adults to live independently. However, technology adoption by older adults, particularly assistive technology (AT), is limited and little is known about the types of AT used among older adults. This study explored the use of key information and communication technologies (ICT) and AT among community-dwelling adults aged ≥ 65.

**Methods:**

A cross-sectional study was conducted among community-dwelling adults aged ≥ 65 in southern Germany using a paper-based questionnaire. The questionnaire included questions on the three domains sociodemographic aspects, health status, and technology use. Technology use was considered separately for key ICT (smartphone, computer/laptop, and tablet) and a range of 31 different AT. Data were analyzed using descriptive statistics, univariate analyses, and Bernoulli Naïve Bayes modelling.

**Results:**

The questionnaire was answered by 616 participants (response rate: 24.64%). ICT were used by 497 (80.68%) participants and were associated with lower age, higher level of education, living together with someone, availability of internet connection, higher interest in technology, and better health status (*p* < .05). No association was found with sex and size of the hometown. The most frequently owned AT were a landline phone, a body scale, and a blood pressure monitor. Several AT related to functionality, (instrumental) activities of daily living- (IADL), and morbidity were used more frequently among non-ICT users compared to ICT-users: senior mobile phone (19.33% vs. 3.22%), in-house emergency call (13.45% vs. 1.01%), hearing aid (26.89% vs. 16.7%), personal lift (7.56% vs. 1.61%), electronic stand-up aid (4.2% vs. 0%). Those with higher interest in technology reported higher levels of benefit from technology use.

**Conclusions:**

Despite the benefits older adults can gain from technology, its use remains low, especially among those with multimorbidity. Particularly newer, more innovative and (I)ADL-related AT appear underutilized. Considering the potential challenges in providing adequate care in the future, it may be crucial to support the use of these specific AT among older and frailer populations. To focus scientific and societal work, AT with a high impact on autonomy ((I)ADL/disease-related) should be distinguished from devices with a low impact on autonomy (household-/ comfort-related).

**Supplementary Information:**

The online version contains supplementary material available at 10.1186/s12889-023-16926-8.

## Background

The demographic landscape is undergoing a significant shift towards an aging population, increasing the need for care in the future. Additional challenges arise from a shortage of skilled healthcare professionals and growing healthcare expenses [1]. Thus, reducing morbidity and the demand for care is essential for the continued stability of healthcare systems. Policy makers are trying to prioritize aging in place and home care over institutionalization whenever possible [2, 3]. Technology can be one way to enable independent living, improve quality of life, and promote healthy, active aging [4–8].

Especially those living with multimorbidity or frailty might benefit from technological assistance in their daily life. Information and communication technologies (ICT) and assistive technologies (AT) aim to compensate impairments related to diseases, disabilities, or old age and support independence of older adults. A rapidly increasing body of literature reports the effectiveness of a wide variety of ICT and AT for older adults with different impairments and diseases such as dementia and mild cognitive impairment [9, 10], Parkinson’s Disease [11–13], or chronic obstructive pulmonary disease [14, 15].

Smartphones, computers, and tablets are considered key ICT that are omnipresent in todays connected world and an essential part of active participation in the community [16]. AT are manifold and heterogenous with different levels of complexity and price ranges [17]. Prominent examples are wearable devices [5], robotic systems [18], smart in-home technology [7], or mobile health applications [19]. Devices range from more traditional ones such as hearing aids [20] to new and innovative devices such as tremor spoons [13], smart electric walkers [21, 22], or artificial intelligence enhanced vision aids [23]. Other examples include systems that manage fall risk [24, 25], ensure safe and timely medication [26, 27], enable personal disease management [28, 29], or promote social connections [30, 31].

While there clearly is an abundance of available AT for older adults with different frailty levels and diseases, it is uncertain which of those devices, especially the more innovative ones, are being used by the target group in their daily lives. ICT are a prerequisite for the use of many AT (e.g., when a smartphone or tablet application is required for AT operation) and could thus be considered an indicator for AT use [32]. Smartphone use among those aged 65 + has increased from 13% in 2012 to 61% in 2021 but remains below the rates for adults aged 18–49 (95%–96%) [33]. For older adults with increased levels of frailty or multimorbidity, lower numbers have been reported in the past [34]. At the same time, AT uptake among the older community has been low [7]. A study from 2021 reported a prevalence of around 30% for AT supporting hearing, vision, or mobility among healthy Canadians aged 65–85 [20]. However, only more traditional devices have been included and the frequency has not been reported for specific devices. In another study, a low level of wearable use of 17.49% was reported for US Americans aged 65 or older [5]. Taken together, few studies report usage rates for AT among the age group above 65 and there are inconsistencies regarding associated sociodemographic and health-related variables [35]. Thus, the goal of this study was (1) to explore the use of key ICT and associated sociodemographic characteristics and (2) to determine and describe which AT are used among community-dwelling adults aged ≥ 65.

## Methods

A cross-sectional study was conducted in a county in southern Germany among older community-dwelling adults aged ≥ 65. The survey instrument was a paper-based questionnaire sent out via the postal service. An ethics approval was obtained from the Joint Ethics Committee of the Bavarian Universities of Applied Sciences (GEHBa-202,101-V-014, 5 Feb 2021). This article is written in accordance with the Strengthening the Reporting of Observational Studies in Epidemiology (STROBE) Statement (supplementary file 1: STROBE Checklist) [36].

### Study population

Community-dwelling adults aged ≥ 65 and registered at a private residence or a care facility in the selected county were eligible to participate. Based on an estimated frequency of AT in the target population of 20–30% [5, 20] and an expected response rate of 10-15%, we aimed to achieve a sample size of at least 250 for an estimated 95% confidence in the analysis (calculated using the web-based, open source software OpenEpi [37]). Considering we did not send out any reminders or offered compensation, the expected response rate was set lower than has been reported by other cross-sectional studies [38, 39]. Addresses were obtained through regional registration offices covering towns ranging in size from < 1,000 to > 10,000 inhabitants. A randomly selected sample, sized in relation to the ratio of persons aged ≥ 65 registered at each registration office, was requested from all 15 registration offices in the selected county. In total, the individual samples added up to the targeted 2,500 persons (supplementary file 2: Overview of sample compilation). Only names, sex, and addresses were requested and deleted immediately after the questionnaires had been sent out. One registration office denied to release the requested address data.

### Questionnaire and measures

To enable participation of older adults with different ranges of digital literacy, a paper-based questionnaire was developed in German. No personal data was recorded on the questionnaire. The questionnaire included questions on the following three domains: (1) sociodemographic aspects, (2) health status, and (3) technology use.

Sociodemographic variables studied included age, sex, education (highest degree), living situation (alone, with others), size of hometown (< 1,000; 1,000–9,999; ≥10,000), technology interest (no, little, medium, strong interest), and home broadband internet connection (yes, no). Age was categorized into three groups, 65–70, 71–80, and 80+. Education status was categorized as having ≤ 10 years or > 10 of education. Having > 10 years of education is the threshold needed to qualify for university in Germany. Comorbidities were captured using the list from the Cumulative Illness Rating Scale for a geriatric population (CIRS-G) [40]. A new variable was computed categorizing health status in three levels: no comorbidities, 1–2 comorbidities, and ≥ 3 comorbidities [41]. Technology use was reported for two domains: (1) Key ICTs including smartphone, computer/laptop, and tablet (based on the definition in [42]), and (2) different AT covering devices related to single deficits/diseases, (instrumental) activities of daily living ((I)ADL), and comfort or household tasks (Table 1). For ICTs frequency of use (daily, weekly, rarer, never) was also collected. ICT-Users are defined as persons using at least one of the three key technologies (smartphone, laptop/computer, tablet). The AT were selected based on a compilation of the most promising devices for older adults developed by the senior community service of the German city of Hannover [43] and also included devices related to functional deficits, disability and/or frailty, respectively the domains of a comprehensive geriatric assessment (CGA) such as mobility, cognition, and the ability to self-aid [44, 45] (Table 1). Perceived benefit from technology use was captures using three levels: High, medium, little/no use.

**Table 1:** Overview of assistive technologies included in the questionnaire (based on [43])

Single deficit-/ disease-related	(I)ADL^1^- and personal-safety-related	Household-, comfort- and exterior-safety-related
- Blood pressure monitor - Blood sugar monitor - Tremor spoon - Health application - Digital viewing aid - GPS-locating device - Body scale - Hearing aid - Doorbell/ringtone intensifier	- Mobile/ in-house emergency call - Digital calendar - Electronic medication dispenser - Personal lift (e.g., for stairs, bathtub) - Electronic stand-up aid - Wearable device (e.g., smartwatch, fitness tracker, smart clothing) - Sensor mat - Fall detection device - Speech recognition assistant - Senior tablet - Senior mobile phone - Video call application - Electric walker	- Lighting system (e.g., motion detectors, light sensors) - Stove switch-off - Water alarm/ regulator - Landline phone - Universal remote - Support/ household robot - Home automation/ smart home system - Key finder - Door/ window alarm

^1^ (I)ADL: (Instrumental) activities of daily living

The primary outcome was ICT use, defined as using at least one key ICT, i.e., smartphone, tablet, or computer/laptop, or non-use. Secondary outcomes were the frequency of ICT use, the use of different AT, and the benefit gained from the technology used.

### Study procedure

Questionnaires were sent out via the German postal service in the beginning of April 2021. Envelopes included a cover letter explaining the study goals and potential risks and a stamped return envelope with the return address printed on. Informed consent was obtained from all participants. Returned questionnaires were accepted until June 15th, 2021. No reminders were sent out.

### Data analysis

Using the QuestorPro software (Blubbsoft GmbH) the questionnaire was transferred to a machine-readable format prior to distribution. All returned questionnaires were checked for inconsistencies or error-prone markings. Nine of the returned questionnaires were recorded manually and checked by a second researcher as they contained handwritten information in the margins and/or difficult to determine checkmarks. All other questionnaires were automatically recorded by scanning the documents and using the software for data verification and extraction. Free text was recognized but had to be entered manually into the database. The entered data was verified by a second person using random samples.

Descriptive statistics were calculated as mean and standard deviation for numerical variables and as frequencies and percentages for categorical variables. Pearson’s chi-square test and Fisher’s exact test were used to analyze categorical variables. The statistical significance was set at p < .05 for all tests. Data analysis and visualization was done using R Version 4.1.2 on R Studio Version 2023.03.1 and Microsoft Excel Version 2304 for Windows. In addition, we calculated a Bernoulli Naive Bayes Model with Laplace smoothing [46] to see if there is a general trend between the AT and the benefit rating of the technology. All subjects that gave a technology benefit rating were included in the analysis, irrespective of ICT-use. Only AT with > 5 reported users in our sample were analyzed. For the calculation, we used the scikit-learn 1.2.2 library for python [47], with the default hyperparamters (alpha = 1.0, force_alpha = False, binarize = 0.0, fit_prior = True, class_prior = None=). Model performance was evaluated by calculating the accuracy score and the receiver operator characteristics (ROC) area under the curve (AUC), the latter using the one-vs-rest method calculating the micro average. The 95% confidence intervals (CI) were determined by bootstrapping with 1000 iterations [48]. To examine whether specific devices are associated with the overall benefit rating of the technology, we extracted the conditional probability of the AT (given the benefit rating) from the model using the ‘feature_log_prob_’ attribute. For better comparability, we applied a softmax function to these probabilities.

## Results

Six hundred and nineteen participants returned the questionnaire (response rate: 24.76%). Data are presented for 616 participants who reported on ICT use. Table 2 shows the participant characteristics overall and stratified by ICT use. The mean age was 74.22 (± 7.03) with a range from 65 to 101. Sex was distributed equally with 301 (50.08%) female participants. Approximately one fourth (n = 148, 24.83%) of the participants had > 10 years of education. More than half of the participants (n = 310, 52.19%) lived in cities/villages below 10.000 inhabitants. Strong interest in technology was recorded by 121 (19.71%) participants.

**Table 2:** Characteristics of the study population by ICT-users and non-ICT-users

	Total (n = 616; 100%)		ICT-users (n = 497; 80.68%)		Non-ICT-users (n = 119; 19.32%)		*P*
	n(%)	na	n(%)	na	n(%)	na	
**Age (years)**		1		1		0	< .001^1^*
mean (SD)	74.22 (7.03)		72.74 (5.97)		80.35 (7.8)		
65–70	237(38.54)		220(44.35)		17(14.29)		
71–80	252(40.98)		210(42.34)		42(35.29)		
80+	126(20.49)		66(13.31)		60(50.42)		
**Sex**		15		12		3	.185^1^
Female	301(50.08)		236(48.66)		65(56.03)		
Male	300(49.92)		249(51.34)		51(43.97)		
**Education**		20		15		5	< .001^1^*
≤ 10years	448(75.17)		344(71.37)		104(91.23)		
> 10 years	148(24.83)		138(28.63)		10(8.77)		
**Living situation**		1		1		0	.008^1^*
Alone	161(26.18)		118(23.79)		43(36.13)		
With others	454(73.82)		378(76.21)		76(63.87)		
**Size of hometown**		22		17		5	.45^1^
< 1,000	51(8.59)		38(7.92)		13(11.4)		
1,000–9,999	259(43.6)		209(43.54)		50(43.86)		
≥ 10,000	284(47.81)		233(48.54)		51(44.74)		
**Internet connection**		7		5		2	< .001^1^*
Yes	524(86.04)		474(96.34)		50(42.74)		
No	85(13.96)		18(3.66)		67(57.26)		
**Technology interest**		2		2		0	< .001^2^*
Strong	121(19.71)		118(23.84)		3(2.52)		
Medium	327(53.26)		304(61.41)		23(19.33)		
Little	119(19.38)		67(13.54)		52(43.7)		
Not at all	47(7.65)		6(1.21)		41(34.45)		
**Health status**		0		0		0	.008^2^*
No comorbidities	46(7.47)		43(8.65)		3(2.52)		
1–2 comorbidities	247(40.1)		206(41.45)		41(34.45)		
≥ 3 comorbidities	323(52.44)		248(49.9)		75(63.03)		

^1^Calculated using Pearson’s Chi-square test

^2^Calculated using Fisher’s Exact Test

^*^p < .05

### ICT use

In this study, 497 (80.68%) users and 119 (19.32%) non-users of ICT were observed. ICT-users were noted to be younger, have higher levels of education, live more often together with others, have internet connection, to have a higher interest for technology, and to have on average less comorbidities than non-ICT-users.

There were 425 (85.51%) smartphone users, 421 (84.71%) computer users, and 220 (44.27%) tablet users. Of all ICT-users, 101 (20.32%) reported using one device, 223 (44.87%) reported using two devices, and 173 (34.81%) reported using three devices. For those using only one device, a computer was the most frequent one overall. Frequency of use, however, was highest for smartphones. Daily smartphone use was at 66.67% for single device users but rose above 80% for multiusers compared to 46%–63.64% for computer users.

**Fig. 1:**
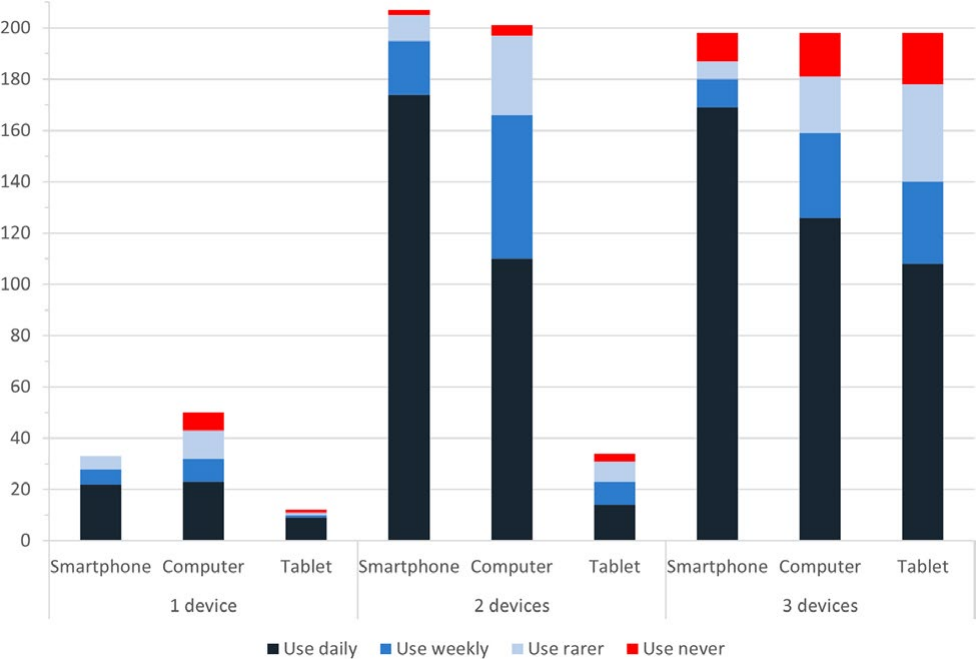
Frequency of device use by number of ICT devices owned with the number of participants on the y-axis

There was a discrepancy between ICT ownership and ICT use. Among the participants, 13 (3.06%) smartphone owners, 28 (6.65%) computer owners, and 24 (10.91%) tablet owners did not use their devices (“use never”). While participants who only owned a smartphone always used it at least sometimes, participants who owned only a computer did not use it in 14% (n = 7) of the cases. Ownership was reported by 95 (19.11%) participants for one device, by 221 (44.47%) participants for two devices, and by 198 (39.84%) participants for three devices. Figure 1 presents frequency of device use (incl. “use never”) by number of ICT devices owned.

### AT use

**Fig. 2:**
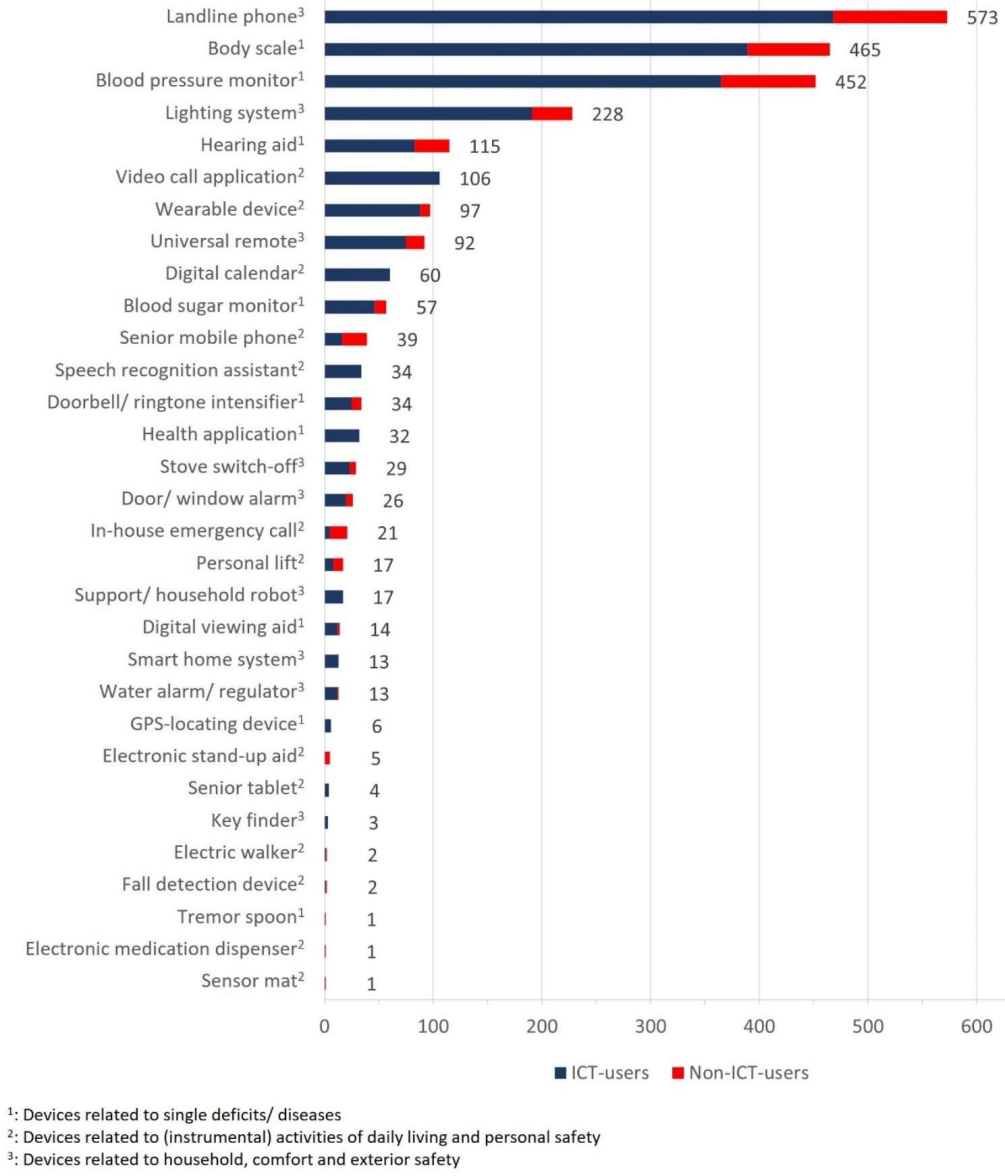
Total use of AT by ICT-users and non-ICT-users

AT use has been recorded for 31 different devices or device categories (Table 1). The most frequently used device was a traditional landline phone (n = 573, 93.02%), followed by a body scale (n = 465, 75.49%), and a blood pressure monitor (n = 452, 73.38%) (Fig. 2). A sensor mat, a digital pill dispenser, and a tremor spoon were only named once respectively, all three by non-ICT-users.

Several devices were exclusively used by ICT-users, namely a video call application, a digital calendar, a speech recognition assistant, a health application, a support/household robot, a GPS locating device, a smart-home system, a senior tablet, and a key finder. The following AT were used with a higher ratio among non-ICT-users compared to ICT-users: a senior mobile phone (19.33% vs. 3.22%), an in-house emergency call (13.45% vs. 1.01%), a hearing aid (26.89% vs. 16.7%), a personal lift (7.56% vs. 1.61%), and an electronic stand-up aid (4.2% vs. 0%).

**Fig. 3:**
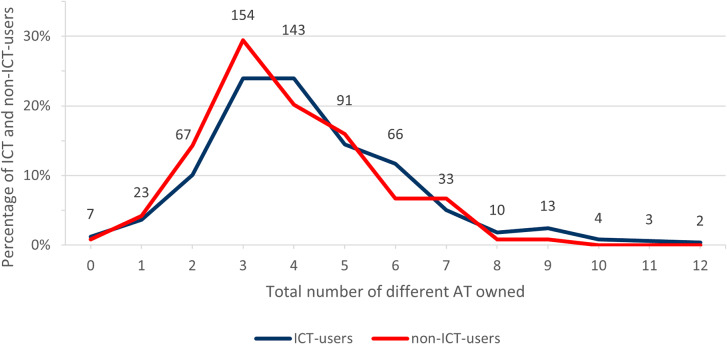
Distribution of the total number of different AT owned by ratio of ICT-user vs. non-ICT-users. Numbers above the lines indicate the sum of ICT-users and non-ICT-users using the respective amount of different AT depicted on the x-axis

Participants owned between zero and 12 different AT. The amount of owned AT within our study population showed a right skewed distribution with a mode of 3 and a median of 4 devices. Seven (1.14%) participants owned no AT and nine (1.5%) participants owned ≥ 10 devices. While all participants owning ≥ 10 AT were exclusively ICT-users, overall, no significant difference was found regarding ICT-use and the number of AT devices owned (Fig. 3).

### Technology benefit

There were 476 (77.27%) participants who reported their perceived overall benefit from the used technology (ICT and AT combined). Most people indicated a high benefit (n = 287, 60.29%), followed by medium (n = 154, 32.35%), and little or no benefit (n = 35, 7.35%). There was a significant difference between ICT-users and non-ICT-users (p < .001). Only about 4.15% of ICT-users reported low or no benefit gained from the used technology. In contrast, among non-ICT-users this rating was more frequent, with 27.27% reporting low or no benefit. High benefit was reported by users of more innovative AT such as smart home systems, support/household robots or health applications.

**Fig. 4:**
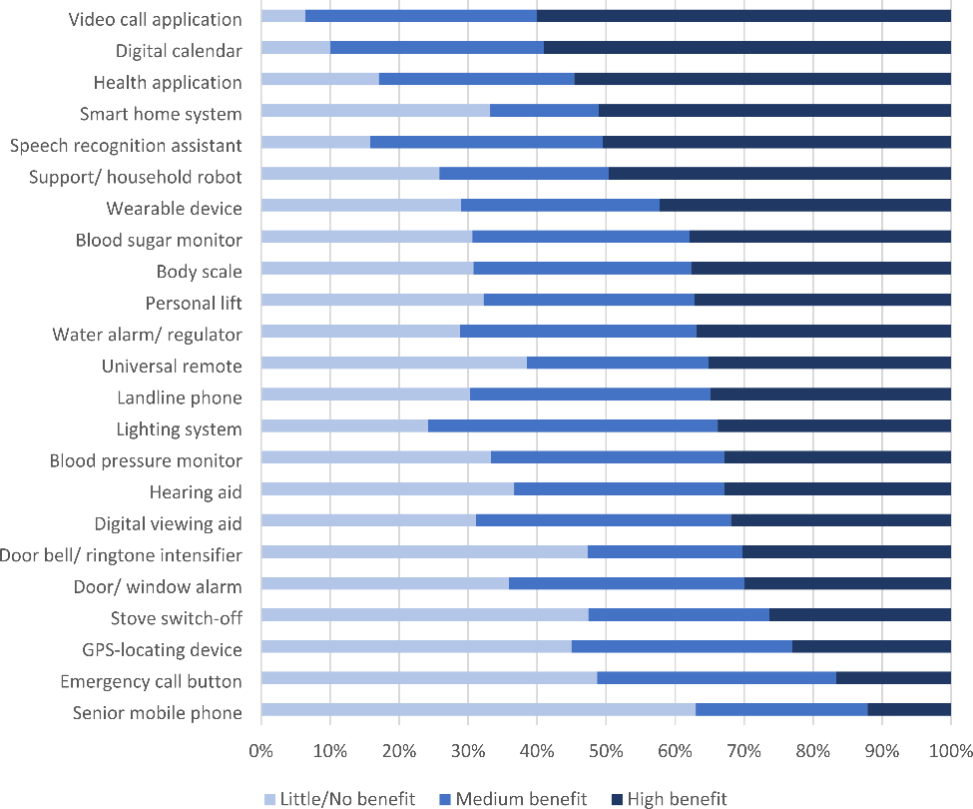
Association of the technology benefit rating with the relative use of different AT. All subjects that gave a technology benefit rating were included, irrespective of ICT-use. Only AT with n > 5 reported users in our sample were included in the analysis. Displayed are the empirical probabilities for an AT given the technology benefit rating after applying a softmax function

The Bernoulli Naïve Bayes analysis could estimate the technology benefit rating from the presence or absence of owned AT with an accuracy of 0.62 (95% CI 0.58–0.67) and an ROC AUC of 0.82 (95% CI 0.8–0.84). The empirical probabilities for presence or absence of an AT (given the technology benefit rating) after applying a softmax function are presented in Fig. 4, which can be understood as displaying the predictive power of the technology benefit rating for the relative use of different AT. Little or no technology benefit was inversely related to the use of a video call application, a digital calendar, a health application, and a speech recognition assistant. In other words, comparatively few people who reported little or no benefit from technology used these devices. In turn, a high technology benefit was inversely related to the use of a senior mobile phone and an emergency call button.

## Discussion

ICT and AT have the potential to improve independence, increase safety, and help users stay socially connected [49, 50]. These aspects are especially important for older adults and high hopes are being put in the use of technology to assist healthcare providers [17]. This study presents a comprehensive overview of technology use among older, community-dwelling adults for both key ICT (smartphone, computer/laptop, and tablet) and a range of different AT, including those specifically for frailer older adults.

Regarding ICT-use, it could be shown that younger age, higher level of education, living together with others, the availability of an internet connection, higher interest in technology, and a better health status are associated characteristics. Other studies with older adults had similar results and identified higher education, higher income level, and better overall health status to be associated with an increased likelihood of technology use among older adults [5, 34]. No association could be found for the size of the hometown. This is in contrast to a recent study from the United States that found that older adults who live in rural areas tend to use less technology [30]. One explanation could be that the population density is much higher in Germany and even rural areas are usually well accessible. Other studies showed that female gender and living alone increased the likelihood for technology uptake, contradicting the results presented here [5, 51]. A recent review confirms these apparent inconsistencies within the current research, stating that the influence of sociodemographic variables and health condition on technology uptake remains unclear [35].

Overall, ICT-use or non-use was not significantly associated with the number of different AT owned. However, similarly to other research, the association was significant when looking at individual devices or device groups [32]. The present study confirmed that non-ICT-users are exempt from many potentially beneficial AT that rely on a smartphone or other mobile device. Examples are video call systems, health applications (used to e.g., monitor chronic diseases), digital calendars, smart home systems, GPS locating devices, or speech recognition assistance systems. Perceiving the overall technology benefit as high was associated with the ownership of many of these devices. Likewise, estimating the overall technology benefit rating from AT use in a Bayesian Model yielded good results (ROC AUC 0.82). There were some AT for which the usage rate was higher among non-ICT-users such as an in-house emergency call, a hearing aid, or a personal lift. These devices are related to functional deficits and multimorbidity which is consistent with the results that non-ICT-users are older and have more comorbidities. Interestingly, a senior mobile phone was used about six times as frequently among non-ICT-users compared to ICT-users. This suggests that non-ICT users have a desire to remain connected and engaged but might not feel comfortable with using a smartphone.

About 96% of ICT-users reported high or medium perceived benefit gained from using technology, confirming the potential and advantages of technology use. However, across the entire study population, including ICT-users, AT remained largely underused. Classic devices such as a landline phone, a body scale, and a blood pressure monitor remain the most used and newer, more innovative technologies lack behind [7]. While existing studies show, that older adults are generally willing to use technology [2], several aspects can explain the current trend. First, AT, particularly modern and innovative ones, can arouse distrust regarding functionality and privacy. Also, many devices are not developed with the target group in mind, reducing usability [52, 53]. Second, technological devices can be expensive, and many older adults may not be able to afford them [54]. Most AT are not (yet) covered by insurance or other forms of financial assistance, keeping them out of reach for many individuals. Third, while the market offers an abundance of AT solutions, many devices are only available online and service providers or healthcare professionals lack the knowledge to give appropriate recommendations [55]. This gap in availability and knowledge limits access to AT for many older adults. Last, the use of AT can raise concerns about social isolation and dependency. Some older adults may feel that using modern AT makes them more reliant on others for technological assistance, which can be detrimental to their self-esteem and overall well-being [54, 56]. For many AT, the numbers reported in this study were too small and larger sample sizes are needed to conduct conclusive analyses. However, it has been found that factors that affect adoption of technology might be the same across different types of devices [35].

**Fig. 5:**
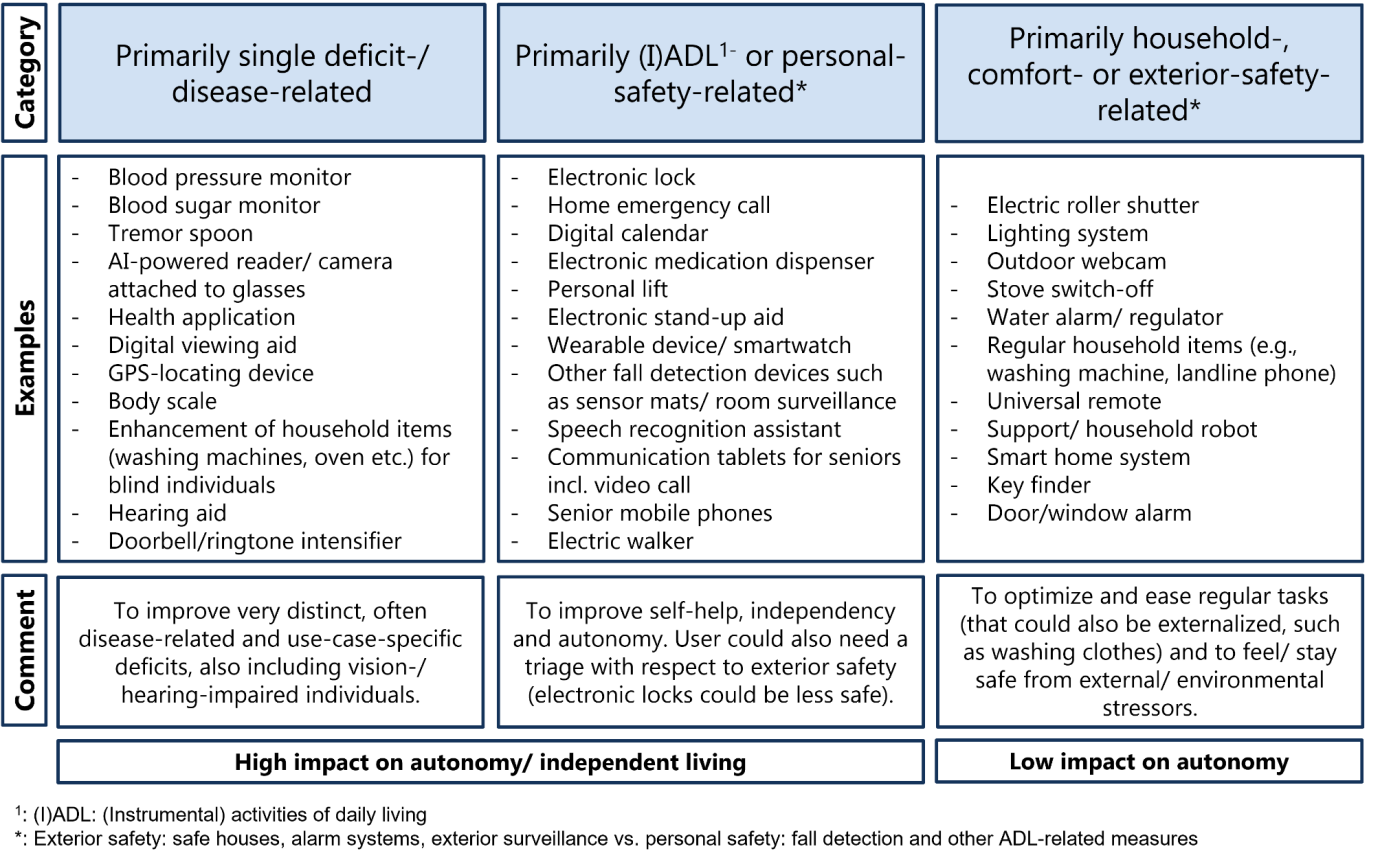
Proposal for separation of AT into three categories with different goals and different levels of impact on autonomy and independent living

Around 3% of participants (n = 18) also added household items, standard entertainment devices, or sports equipment as AT they use in their daily life, highlighting the issue that the term assistive technology is a very broad umbrella term that makes homogeneous research difficult [17]. A 2015 definition by the World Health Organization states that AT include “any item, piece of equipment or product, whether it is acquired commercially, modified or customized, that is used to increase, maintain or improve the functional capabilities of individuals with disability” [57, p.14] and thus confirms this heterogeneity. We believe that there is a relevant difference between AT that are developed to improve autonomy and independence versus those that are well established and primarily related to exterior safety or comfort. For a more focused scientific and societal appraisal of these devices, we propose to highlight items that are associated with use-cases related to (I)ADL or clear deficits/ diseases (high impact on autonomy) and to separate them from devices that are closer linked to household tasks, comfort, and exterior safety (low impact on autonomy) (Fig. 5).

As an example, a patient suffering from diabetes but without further restrictions does not have the same needs as a person suffering from diabetes who also has polyneuropathy and mobility, visual, and hearing impairments. Both use-cases could however, benefit from AT in terms of autonomy and independent living. An external webcam, a landline-phone, or automatic lighting are much less relevant for independent living and more comfort related [58]. A CGA provides a multidisciplinary view of the patient and could help to identify the most promising AT for an individual by separately evaluating ADL and IADL, mobility, emotion including loneliness, nutrition, social network, and others [58]. There are different examples that can be mentioned: lonely people could be offered easy to use senior phones or tablets, people with IADL difficulties could be further evaluated for electronic medication dispensers, prefilled by caregivers, or people with frequent falls could use wearables with fall detection. Thus, we suggest considering the domains of a CGA when categorizing different AT to identify all deficits and resources of an individual and enhance understanding, and ultimately use of AT among the target population. In case it is not possible to perform a full CGA, other screening tools focusing for example on frailty (such as the Clinical Frailty Scale [59]), ADL (such as the Barthel Index [60]), or increased risk (such as the Identifying Seniors at Risk (ISAR) score [61, 62]) could be used.

With almost 25%, the response rate for this study was higher than expected considering that no reminders or incentives were given. This might have been due to a general interest in the topic. Another explanation might be the restrictions due to the Covid-19 pandemic that caused more people to be at home with spare time on their hands. Other cross-sectional studies with older participants reported response rates between 40 and 60%, but made use of interventions to boost responses (such as sending out reminders [38, 63, 64] or cooperating with doctor’s offices [65]).

### Limitations

For this study, addresses were only obtained from one county in Southern Germany. A regional bias in the data is possible and generalizability might be limited to predominantly rural areas in countries with similar economic status and level of digitalization as Germany. Additionally, the second largest registration office denied our request for address data. Older adults with severe physical or cognitive impairments are less likely to participate, resulting in a potential sample bias. Additionally, no pre-selection was made regarding sex. As there are more female citizens in the age group ≥ 65, a potential oversampling of female participants is likely. The questionnaire used was developed by the research team and did not undergo a validation process. The questionnaire was not tested in a rigorous manner. Possible misunderstandings with respect to the definition of an AT may explained the heterogeneity in the answers. Furthermore, there is no information available on how participants answered the questionnaire (e.g., with the help from a relative). As the questionnaires were returned anonymously, it can also not be determined if the person, the questionnaire was addressed to, was the one answering the questions or if the document was handed to the partner or someone else. Last, as this is a cross-sectional study, no causal relationships can be determined from the analysis.

## Conclusion

This study presented insights into the use of key ICT as well as standard and technologically advanced AT among older, community-dwelling adults. While most older adults aged ≥ 65 use some type of technology, newer, more innovative AT appear underutilized. Additionally, the use of AT related to functional deficits (IADL) and to certain diseases (morbidity), remains low, while more ordinary household devices and ICT are highly utilized. However, for those who do use technology, the perceived benefits are high. To find a common language and to categorize these tools for research purposes among frail older people we propose a categorization to separating disease-, IADL- and personal safety-related devices with higher potential impact for autonomy from exterior safety-/ comfort-related AT and established household-related items with a lower impact.

Research and development that focuses especially on the first two categories could leverage independent living in challenging situations. By providing insight into the characteristics associated with the use or non-use of ICT and AT, this study can assist community workers and policy makers with targeting relevant information and specific programs.

### Electronic supplementary material

Below is the link to the electronic supplementary material.

Supplementary file 1: STROBE Checklist

Supplementary file 2: Overview of sample compilation

## Data Availability

The datasets used and/or analyzed during the current study are available from the corresponding author on reasonable request.

## Abbreviations

AT: Assistive technology
AUC: Area under the curve
CGA: Comprehensive geriatric assessment
CI: Confidence Interval
(I)ADL: (Instrumental) activities of daily living
ICT: Information and communication technology
ROC: Receiver operator characteristics
STROBE: Strengthening the Reporting of Observational Studies in Epidemiology

## References

[1] Beard JR, Officer A, de Carvalho IA, Sadana R, Pot AM, Michel JP (2016). The World report on ageing and health: a policy framework for healthy ageing. Lancet.

[2] Jaschinski C, Ben Allouch S, Peters O, Cachucho R, van Dijk JAGM (2021). Acceptance of Technologies for Aging in Place: a conceptual model. J Med Internet Res.

[3] Soar J, Seo Y, Health, Aged Care Enabled by Information Technology (2007). Ann N Y Acad Sci.

[4] Pedrozo Campos Antunes T, Souza Bulle de Oliveira A, Hudec R, Brusque Crocetta T, Ferreira de Lima Antão JY, de Almeida Barbosa RT (2019). Assistive technology for communication of older adults: a systematic review. Aging Ment Health.

[5] Chandrasekaran R, Katthula V, Moustakas E (2021). Too old for technology? Use of wearable healthcare devices by older adults and their willingness to share health data with providers. Health Inf J.

[6] Walsh RJ, Lee J, Drasga RM, Leggett CS, Shapnick HM, Kottorp AB (2020). Everyday Technology Use and overall needed assistance to function in the Home and Community among Urban older adults. J Appl Gerontol.

[7] Dermody G, Fritz R, Glass C, Dunham M, Whitehead L (2021). Factors influencing community-dwelling older adults’ readiness to adopt smart home technology: a qualitative exploratory study. J Adv Nurs.

[8] Wiles JL, Leibing A, Guberman N, Reeve J, Allen RE (2012). The meaning of aging in place to older people. Gerontologist.

[9] Howard R, Gathercole R, Bradley R, Harper E, Davis L, Pank L (2021). The effectiveness and cost-effectiveness of assistive technology and telecare for independent living in dementia: a randomised controlled trial. Age Ageing.

[10] Nishiura Y, Nihei M, Nakamura-Thomas H, Inoue T (2021). Effectiveness of using assistive technology for time orientation and memory, in older adults with or without dementia. Disabil Rehabilitation: Assist Technol.

[11] Ginis P, Heremans E, Ferrari A, Dockx K, Canning CG, Nieuwboer A (2017). Prolonged walking with a Wearable System Providing Intelligent Auditory Input in people with Parkinson’s Disease. Front Neurol.

[12] Lakshminarayana R, Wang D, Burn D, Chaudhuri KR, Galtrey C, Guzman NV (2017). Using a smartphone-based self-management platform to support medication adherence and clinical consultation in Parkinson’s disease. NPJ Parkinsons Dis.

[13] Ryden LE, Matar E, Szeto JYY, Hammond DA, Clouston P, Lewis SJG (2020). Shaken not stirred: a Pilot Study Testing a Gyroscopic spoon stabilization device in Parkinson’s Disease and Tremor. Ann Indian Acad Neurol.

[14] Infarinato F, Jansen-Kosterink S, Romano P, van Velsen L, Op den Akker H, Rizza F (2020). Acceptance and potential impact of the eWALL platform for Health Monitoring and Promotion in persons with a chronic disease or age-related impairment. Int J Environ Res Public Health.

[15] McCabe C, McCann M, Brady AM (2017). Computer and mobile technology interventions for self-management in chronic obstructive pulmonary disease. Cochrane Database of Systematic Reviews.

[16] Malinowsky C, Nygård L, Tanemura R, Nagao T, Noda K, Nakata O (2018). Everyday technology use among older adults in Sweden and Japan: a comparative study. Scand J Occup Ther.

[17] de Witte L, Steel E, Gupta S, Ramos VD, Roentgen U (2018). Assistive technology provision: towards an international framework for assuring availability and accessibility of affordable high-quality assistive technology. Disabil Rehabilitation: Assist Technol.

[18] Shishehgar M, Kerr D, Blake J (2019). The effectiveness of various robotic technologies in assisting older adults. Health Inf J.

[19] Khoong EC, Olazo K, Rivadeneira NA, Thatipelli S, Barr-Walker J, Fontil V (2021). Mobile health strategies for blood pressure self-management in urban populations with digital barriers: systematic review and meta-analyses. NPJ Digit Med.

[20] Ishigami Y, Jutai J, Kirkland S (2021). Assistive device use among Community-Dwelling older adults: A Profile of Canadians using hearing, Vision, and mobility Devices in the canadian longitudinal study on aging. Can J Aging / La Revue Canadienne du Vieillissement.

[21] Martins M, Santos C, Frizera A, Ceres R (2015). A review of the functionalities of smart walkers. Med Eng Phys.

[22] Sato W, Tsuchida Y, Li P, Hasegawa T, Yamada Y, Uchiyama Y. Identifying the Effects of Assistive and Resistive Guidance on the Gait of Elderly People using a Smart Walker. 2019 IEEE 16th International Conference on Rehabilitation Robotics (ICORR). 2019;198–203. 10.1109/icorr.2019.8779556. 10.1109/ICORR.2019.8779556 31374630

[23] Celik N, Rohrschneider K (2018). Elektronische Hilfsmittel - Neue Möglichkeiten zur Rehabilitation Sehbehinderter. Der Ophthalmologe.

[24] Lauriks S, Meiland FJM, Osté JP, Hertogh C, Dröes R-M (2020). Effects of Assistive Home Technology on quality of life and falls of people with dementia and job satisfaction of caregivers; results from a pilot randomized controlled trial. Assist Technol.

[25] Moore K, O’Shea E, Kenny L, Barton J, Tedesco S, Sica M (2021). Older adults’ experiences with using Wearable Devices: qualitative systematic review and Meta-synthesis. JMIR mHealth and uHealth.

[26] Lam AY, Nguyen JK, Parks JJ, Morisky DE, Berry DL, Wolpin SE (2017). Addressing low health literacy with talking pill bottles: a pilot study in a community pharmacy setting. J Am Pharm Assoc.

[27] Ping Y, Visaria A, Suppiah SD, Tan YW, Malhotra R (2022). Prevalence and correlates of medication reminder app ‘use and use intention’ among older adults. Explor Res Clin Soc Pharm.

[28] Bezerra Giordan L, Tong HL, Atherton JJ, Ronto R, Chau J, Kaye D (2022). The Use of Mobile apps for heart failure Self-management: systematic review of experimental and qualitative studies. JMIR Cardio.

[29] Wang H, Zhao Y, Yu L, Liu J, Zwetsloot IM, Cabrera J (2020). A Personalized Health Monitoring System for Community-Dwelling Elderly People in Hong Kong: design, implementation, and evaluation study. J Med Internet Res.

[30] Byrne KA, Anaraky RG, Dye C, Ross LA, Chalil Madathil K, Knijnenburg B (2021). Examining rural and racial disparities in the relationship between loneliness and Social Technology Use among older adults. Front Public Health.

[31] Latikka R, Rubio-Hernández R, Lohan ES, Rantala J, Nieto Fernández F, Laitinen A (2021). Older adults’ loneliness, social isolation, and physical information and Communication Technology in the era of ambient assisted living: a systematic literature review. J Med Internet Res.

[32] Arthanat S, Wilcox J, Macuch M. Profiles and predictors of Smart Home Technology Adoption by older adults. OTJR (Thorofare N J). 2019;39(4):247–56. 10.1177/1539449218813906. 10.1177/1539449218813906 PMC7042636 30477397

[33] Faverio M. Share of those 65 and older who are tech users has grown in the past decade: Pew Research Center; 2022 [16 Jun 2022]. Available from: https://www.pewresearch.org/fact-tank/2022/01/13/share-of-those-65-and-older-who-are-tech-users-has-grown-in-the-past-decade/.

[34] Smith A, Pew Research Center. Older Adults and Technology Use. ; 2014. Available from: https://www.pewresearch.org/internet/2014/04/03/older-adults-and-technology-use/. Accessed 16 Dec 2022.

[35] Kavandi H, Jaana M (2020). Factors that affect health information technology adoption by seniors: a systematic review. Health Soc Care Commun.

[36] von Elm E, Altman DG, Egger M, Pocock SJ, Gøtzsche PC, Vandenbroucke JP (2007). The strengthening the reporting of Observational Studies in Epidemiology (STROBE) statement: guidelines for reporting observational studies. The Lancet.

[37] Sullivan KM, Dean A, Soe MM (2009). OpenEpi: a web-based epidemiologic and statistical calculator for public health. Public Health Rep.

[38] Van Woerden HC, Angus N, Kiparoglou V, Atherton I, Leung J (2021). Long-term conditions in older people are linked with loneliness, but a sense of coherence buffers the adverse Effects on Quality of Life: a cross-sectional study. J Multidisciplinary Healthc.

[39] Abrahamsen R, Svendsen MV, Henneberger PK, Gundersen GF, Torén K, Kongerud J (2016). Non-response in a cross-sectional study of respiratory health in Norway. BMJ Open.

[40] Parmelee PA, Thuras PD, Katz IR, Lawton MP (1995). Validation of the cumulative illness rating scale in a geriatric residential population. J Am Geriatr Soc.

[41] Davies SJ, Phillips L, Naish PF, Russell GI (2002). Quantifying comorbidity in peritoneal dialysis patients and its relationship to other predictors of survival. Nephrol Dialysis Transplantation.

[42] Wallcook S, Nygård L, Kottorp A, Malinowsky C (2021). The use of everyday information communication technologies in the lives of older adults living with and without dementia in Sweden. Assist Technol.

[43] Landeshauptstadt Hannover. Handbuch “Wohnen mit technischer Unterstützung: Geräte, Einsatzfelder, Kosten”. Hannover, GER: Kommunaler Seniorenservice Hannover, Fachbereich Senioren. ; 2015. Available from: https://www.hannover.de/Leben-in-der-Region-Hannover/Soziales/Senioren/Wohnen-im-Alter/Wohnen-mit-technischer-Unterst%C3%BCtzung. Accessed Oct 15 2021.

[44] Deutsche Gesellschaft für Geriatrie. S1-Leitlinie Geriatrisches Assessment der Stufe 2, Living Guideline. AWMF. ; 2022 Mar 27 2023. Available from: https://register.awmf.org/de/leitlinien/detail/084-002LG. Accessed Mar 27 2023.

[45] Parker SG, McCue P, Phelps K, McCleod A, Arora S, Nockels K (2017). What is Comprehensive Geriatric Assessment (CGA)? An umbrella review. Age Ageing.

[46] Manning CD, Raghavan P, Schütze H. Introduction to Information Retrieval. Cambridge University Press; 2008.

[47] Pedregosa F, Varoquaux G, Gramfort A, Michel V, Thirion B, Grisel O (2018). Scikit-learn: machine learning in Python. J Mach Learn Res.

[48] Efron B, Tibshirani RJ. An Introduction to the Bootstrap. 1994:456. 10.1201/9780429246593.

[49] Delello JA, McWhorter RR (2017). Reducing the Digital divide:connecting older adults to iPad Technology. J Appl Gerontol.

[50] Hirmas-Adauy M, Olea A, Matute I, Delgado I, Aguilera X, Poffald L (2019). Assistive Devices for older adults: a longitudinal study of policy effectiveness, Santiago, Chile, 2014–2016. MEDICC Rev.

[51] Etemad-Sajadi R, Gomes Dos Santos G (2019). Senior citizens’ acceptance of connected health technologies in their homes. Int J Health Care Qual Assur.

[52] Mannheim I, Schwartz E, Xi W, Buttigieg SC, McDonnell-Naughton M, Wouters EJM, et al. Inclusion of older adults in the Research and Design of Digital Technology. Int J Environ Res Public Health. 2019;16(19). 10.3390/ijerph16193718. 10.3390/ijerph16193718 PMC6801827 31581632

[53] Grigorovich A, Kontos P, Jenkins A, Kirkland S (2022). Moving toward the Promise of Participatory Engagement of older adults in Gerotechnology. Gerontologist.

[54] Yusif S, Soar J, Hafeez-Baig A (2016). Older people, assistive technologies, and the barriers to adoption: a systematic review. Int J Med Inform.

[55] Tsertsidis A (2020). Challenges in the provision of digital technologies to elderly with dementia to support ageing in place: a case study of a swedish municipality. Disabil Rehabilitation: Assist Technol.

[56] Wilson J, Heinsch M, Betts D, Booth D, Kay-Lambkin F (2021). Barriers and facilitators to the use of e-health by older adults: a scoping review. BMC Public Health.

[57] Disability and Rehabilitation Team of the World Health Organization. WHO global disability action plan 2014–2021 - better health for all people with disability. Geneva, Switzerland: World Health Organization. ; 2015. Available from: https://www.who.int/publications/i/item/who-global-disability-action-plan-2014-2021. Accessed Feb 2 2023.

[58] Couderc AL, Alexandre A, Baudier A, Nouguerede E, Rey D, Pradel V (2020). Preoperative simplified geriatric assessment in planned hip and knee arthroplasty. Eur Geriatr Med.

[59] Rockwood K, Song X, MacKnight C, Bergman H, Hogan DB, McDowell I (2005). A global clinical measure of fitness and frailty in elderly people. CMAJ: Can Med Association J.

[60] Mahoney FI, Barthel DW (1965). Functional evaluation: the Barthel Index: a simple index of independence useful in scoring improvement in the rehabilitation of the chronically ill. Maryland State Med J.

[61] Warburton RN, Parke B, Church W, McCusker J (2004). Identification of seniors at risk: process evaluation of a screening and referral program for patients aged ≥ 75 in a community hospital emergency department. Int J Health Care Qual Assur.

[62] McCusker J, Bellavance F, Cardin S, Trépanier S, Verdon J, Ardman O (1999). Detection of older people at increased risk of adverse health outcomes after an emergency visit: the ISAR screening tool. J Am Geriatr Soc.

[63] Dong H-J, Larsson B, Dragioti E, Bernfort L, Levin L-Ã, Gerdle B (2020). Factors Associated with life satisfaction in older adults with Chronic Pain (PainS65+). J Pain Res.

[64] Ryden AM, Martin JL, Matsuwaka S, Fung CH, Dzierzewski JM, Song Y (2019). Insomnia disorder among older Veterans: results of a Postal Survey. J Clin Sleep Med.

[65] Morris JE, Roderick PJ, Harris S, Yao G, Crowe S, Phillips D (2021). Treatment burden for patients with multimorbidity: cross-sectional study with exploration of a single-item measure. Br J Gen Pract.

