# Supplementary material for: Use and benefit of information, communication, and assistive technology among community-dwelling older adults – a cross-sectional study
Source: BMC Public Health. 2023 Oct 13;23:2004. doi: 10.1186/s12889-023-16926-8 (PMC10576310; doi:10.1186/s12889-023-16926-8)
Supplement: Supplementary file 2 — Overview of sample compilation [file 12889_2023_16926_MOESM2_ESM.docx]

Supplementary file 2: Overview of sample compilation

| Registration office | Total population registered | Population 65+ registered | % of total 65+ | # of addresses requested |
| --- | --- | --- | --- | --- |
| A | 924 | 170 | 0.56% | 14 |
| B | 1,478 | 265 | 0.88% | 22 |
| C | 1,963 | 392 | 1.30% | 33 |
| D | 2,806 | 492 | 1.63% | 41 |
| E | 4,131 | 737 | 2.45% | 61 |
| F | 4,553 | 1,035 | 3.44% | 86 |
| G | 5,175 | 1,039 | 3.45% | 86 |
| H | 7,331 | 1,512 | 5.02% | 126 |
| I | 9,491 | 2,035 | 6.76% | 169 |
| J | 9,677 | 2,258 | 7.50% | 187 |
| K | 13,892 | 2,760 | 9.16% | 229 |
| L | 13,946 | 1,843 | 6.12% | 153 |
| M | 17,687 | 3,700 | 12.29% | 307 |
| N | 23,375 | Request denied | | |
| O | 62,455 | 11,877 | 39.44% | 986 |
| **Total** | **178,884** | **30,115** | **100%** | **2,500** |
